# Beneficial effect of phosphatidylcholine supplementation in alleviation of hypomania and insomnia in a Chinese bipolar hypomanic boy and a possible explanation to the effect at the genetic level

**DOI:** 10.1186/s40064-015-1002-y

**Published:** 2015-05-20

**Authors:** Shitao Rao, Marco H B Lam, Yun Kwok Wing, Larina C L Yim, Winnie C W Chu, Venus S Y Yeung, Mary M Y Waye

**Affiliations:** Croucher Laboratory for Human Genomics, School of Biomedical Sciences, The Chinese University of Hong Kong, Rm324A, Lo Kwee-Seong Integrated Biomedical Sciences Building, Shatin, N.T. Hong Kong; Department of Psychiatry, Shatin Hospital, The Chinese University of Hong Kong, 33 Ah Kong Kok Street, Shatin, N.T. Hong Kong; Department of Imaging and Interventional Radiology, Prince of Wales Hospital, The Chinese University of Hong Kong, Shatin, N.T. Hong Kong

**Keywords:** Phosphatidylcholine supplementation, Metabolism of diacylglycerol, Bipolar hypomania, Diacylglycerol kinase eta (*DGKH*), Susceptibility polymorphism

## Abstract

**Introduction:**

Recent studies indicated that supplementation of phosphatidylcholine has been found to be beneficial for psychiatric diseases and Diacylglycerol Kinase, Eta (DGKH) protein was involved in regulating the metabolism of phosphatidic acid and diacylglycerol. This study reported a case of a 16-year-old Chinese boy with bipolar hypomania symptoms receiving supplementation of phosphatidylcholine, and a genetic study of a risk variant of *DGKH* gene was performed in an attempt to provide an explanation for the potential beneficial effect of phosphatidylcholine supplementation.

**Case description:**

We described a case of a 16-year-old boy with bipolar disorder, who suffered from monthly episodes of insomnia accompanied by hypomania for 5 months despite adherence to medication. After supplementation of phosphatidylcholine, he returned to a normal sleeping pattern and recovered from hypomania symptoms for approximately 14 months. Furthermore, genotyping results showed that this boy carries the risk genotype (G/C) in *DGKH* variant rs77072822 (adjusted *p*-value = 0.025 after 2000 permutation tests).

**Discussion and evaluation:**

The 16-year-old boy appears to have benefited from the supplementation with phosphatidylcholine and recovered from hypomania symptoms. He carries a risk genotype in rs77072822 which lies in the first intron of *DGKH* gene that was mostly reported to be associated with bipolar disorder. Thus, this finding is consistent with the hypothesis that alleviating the phosphatidylcholine deficiencies might accompany with the risk variants of *DGKH* gene, which might improve the efficacies of such supplementation and design new treatment strategies for bipolar disorder.

**Conclusions:**

This study illustrated that a 16-year-old boy with hypomania symptoms responded well to supplementation of phosphatidylcholine and the boy carries a risk genotype in *DGKH* gene for bipolar disorder, which provides a possible explanation for the boy’s beneficial effect at the genetic level.

## Introduction

The use of phosphatidylcholine has been found to be beneficial for the neonatal pathophysiology. Recently Ross et al. had conducted a randomized placebo-controlled clinical trial with 100 healthy pregnant women using supplementation of phosphatidylcholine (Ross et al. 2013). They found that the endophenotypes associated with schizophrenia (i.e. inhibition of the P50 component of the cerebral evoked response to paired sounds) were diminished in subjects taking phosphatidylcholine even when they carry the genetic risk allele for schizophrenia (*CHRNA7* risk allele). Moreover, double-blind placebo-controlled trials of pure lecithin (in a dose of 10 g three times a day) in the treatment of mania also demonstrated improvement of treated compared with placebo in five of the six patients studied (Cohen et al. 1982). A case report of a 13-year-old girl with mania symptoms had also been published with good response to lecithin only while non-responsive to neuroleptics and lithium (Schreier 1982).

The possible mechanism of the beneficial effect of phosphatidylcholine supplementation for schizophrenia or bipolar mania symptoms had been explored by many studies. For example, the number of manic episodes was found to be associated with elevated DNA oxidation in bipolar I disorder (D’Souza et al. 2013). Supplementation of phosphatidylcholine could lead to better transport of vitamin E, which overcome the oxidative damage to activities of the brain (Mesmin and Antonny 2013). Besides, another hypothesis about the unbalance of metabolism of diacylglycerol (DAG) and phosphatidic acid (PA) had been proposed. Since diacylglycerol kinase (DGK) enzymes phosphorylate DAG into PA when receptors in phosphoinositide pathway receiving activation. DAG is released from phospholipids such as phosphatidylcholine (Merida et al. 2008). Both DAG and PA are crucial molecular regulators of a wide range of biological processes. It can be speculated that those individuals with deficient DGK enzymes might not have a balanced DAG and PA, and supplementation with phosphatidylcholine might alleviate a deficiency in the substrate level of PA. Thus, those genes encoding DGK enzymes are likely to play a key role in the pathogenesis of bipolar mania.

Diacylglycerol kinase eta (*DGKH*), encoding the type II DGK enzymes subfamily together with DGK-delta, is involved in regulating the intracellular concentrations of DAG and PA. DGKH protein, encoding by *DGKH,* is a vital protein involved in the lithium-sensitive phosphatidylinositol pathway (Berridge 1989). Moreover, lithium is an effective compound approved by the FDA as a mood-stabilizing drug in the treatment of bipolar mania episodes due to the neurological effects of the ion- Li^+^ in the human body. Therefore, it is believed that *DGKH* might be an important candidate gene associated with bipolar mania symptom based on its biological function.

With the advent of genomics research, it is possible to investigate common genetic variants and see whether any common variant is associated with bipolar disorder. In 2008, *DGKH* was reported to associate with bipolar disorder in a genome-wide association study and many polymorphisms located in *DGKH* were found to be associated in a population (of European origin) as reported by the National Institute of Mental Health or a German replicate sample (Baum et al. 2008), such as rs9532988, rs9532989 and rs1012053. Subsequently, Zeng and colleagues also confirmed that *DGKH* was associated with bipolar disorder in a Chinese Han population including 1139 unrelated bipolar disorder patients and 1138 ethnically matched healthy controls using a tagging single nucleotide polymorphisms (SNPs) strategy (Zeng et al. 2011). In their study, a haplotype block (rs9315885-rs9532988-rs1012053) was identified as a risk haplotype (*P* = 3.87∗10^-6,^ OR = 1.98, 95 % confidence interval = 1.47-2.65). Interestingly, both of the two studies supported that the first intron of *DGKH* containing the top associated polymorphisms or haplotypes is the most significantly associated region with bipolar disorder.

Taken together, it is possible that variants in *DGKH*, especially in the first intron, would be associated with the beneficial effect of phosphatidylcholine supplementation for bipolar mania symptoms. In this study, we reported a bipolar hypomania case of a 16-year-old Chinese boy who had been alleviated of hypomania and insomnia symptoms upon supplementation of phosphatidylcholine. Following this observation, we aimed to investigate whether the potential beneficial effect of phosphatidylcholine supplementation was associated with the fact that the boy carries susceptibility variants in the first intron of *DGKH* for bipolar disorder.

## Materials and methods

### Diagnosis and treatment of a Chinese boy with bipolar hypomania

A 16-year-old boy from Hong Kong Shatin district was diagnosed to suffer from bipolar hypomania symptoms by an experienced clinical psychiatrist in Prince Wales of Hospital (Shatin, Hong Kong) with the assistance of the Chinese-Bilingual Structured Clinical Interview for DSM-IV (Axis I, Patient version) (CB-SCID-I/P) (Spitzer et al. 1990), which is a semi-structured face to face interview to access current and lifetime DSM-IV Axis I diagnosis. The detailed performance of this boy had been documented. Despite his adherence to medication, the boy still experienced monthly episodes of hypomania and sleeplessness after five months post-discharge from the hospital (28th May, 2012). As the subject’s parents felt that their son was already very much sedated and was gaining a lot of weight with the doses of valproate and olanzapine prescribed, and that their son is mostly functional other than the monthly episode of hypomania (which is well tolerable by the family), they did not wish to add more mood stabilizer or atypical antipsychotic medications. The authors chose a patient of 16-year-old as a clinical case since it was a very interesting case with a regular monthly episode of hypomania which is not commonly observed in other bipolar subjects. Since Ross et al. had conducted a randomized placebo-controlled clinical trial using supplementation of phosphatidylcholine successfully (Ross et al. 2013), the boy was put on a daily supplement of one capsule of the supplement (1000 mg) containing the following: phosphatidylcholine 600 mg, Vitamin B1 1 mg, Vitamin B2 1.5 mg, Vitamin B6 1.5 mg, Vitamin B12 1 μg, folate 15 mg, Vitamin E 18 mg (18 IU) (Super Life Phoschol Revival from MJ Life Enterprise Co. Ltd. Taiwan) (Growdon and Wurtman 1980; Hirsch 1991; Lieber 1994; Wurtman et al. 1986). In the month following the supplementation, all of the boy’s hypomania and insomnia symptoms had disappeared.

### Microarray experiment for the Chinese boy and his three family members

Four DNA samples from the 16-year-old Chinese boy and his three healthy family members (mother, father and older sister) were sent to 23andMe to carry out the detection of SNPs using Illumina HumanOmniExpress-24 format chip. 23andMe provides laboratory testing in a CLIA-certified laboratory.

**Fig. 1:**
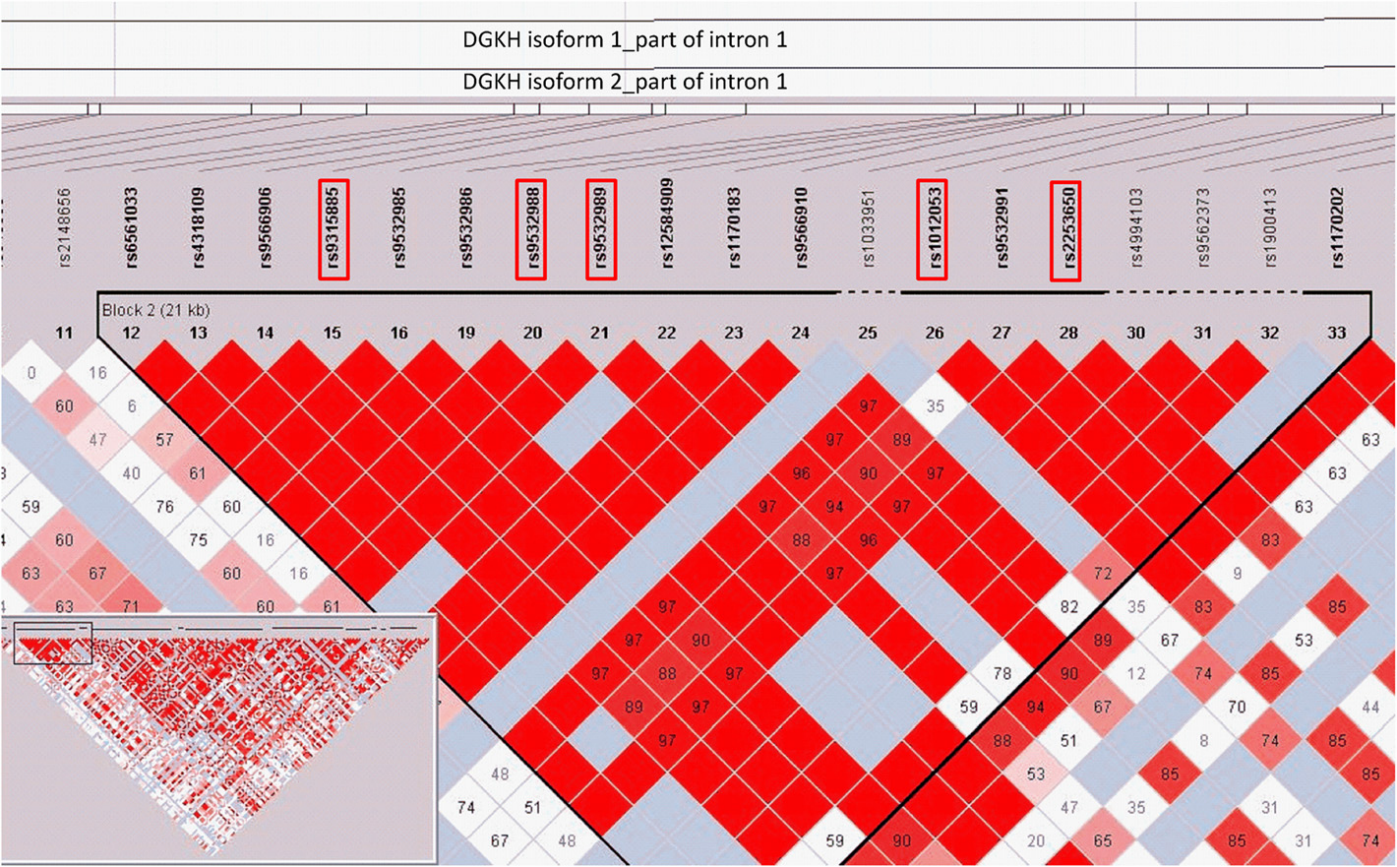
LD Block 2 of the *DGKH* gene. The graph was generated by Haploview program based on the population of Han Chinese genotype data downloaded from the HapMap project (Data Phase III/Rel#2, Feb09). The figure located in the *lower left corner* is the LD Block across the whole *DGKH* gene. The SNPs in the red boxes had been identified to be associated with bipolar disorder in Baum’s study (single marker analyses) or Zeng’s study (haplotype analyses, i.e. rs9315885-rs9532988-rs1012053)

When we focused on the susceptibility region- the first intron of *DGKH* in their sequencing data - there is a “no call” signal for the boy in rs9532989, whereas all of his three family members have signal in this SNP (G/G for his sister and father, G/T for his mother). Thus, we presumed that rs9532989 or the nearby SNPs in the same linkage disequilibrium (LD) block with rs9532989 (Fig. 1) might be associated with the beneficial effect of phosphatidylcholine supplementation for the 16-year-old boy’s hypomania symptoms as these SNPs in the first intron have been associated with bipolar disorder. Therefore, in this study a region covering rs9532989 and its nearby SNPs was chosen for sequencing analyses. This region covers a total of 10 SNPs according to the dbSNP database. They are rs7990452 (A/T), rs9532987 (A/G), rs9562372 (C/T), rs17519207 (C/T), rs77072822 (C/G), rs9532988 (A/G), rs9532989 (G/T), rs79738935 (C/T), rs4142112 (C/G) and rs4142111 (C/T). All of these SNPs were genotyped using the dideoxy-sequencing method after DNA amplification.

### DNA extraction and genotyping

Genomic DNA was extracted from two milliliters of saliva samples of each participant using the Oragene^TM^ DNA self-collection kit according to the manufacturer’s instruction (DNA Genotek, Inc., Ottawa, Canada). After DNA extraction, the concentration of genomic DNA was determined by Nanodrop 2000c spectrophotometer (Thermo Fisher Scientific Inc., MA, US). The 10 SNPs were genotyped using Sanger sequencing in BGI Company after PCR amplification. Briefly, the target region was amplified with the forward primer 5′- AGGCCTTTATGCCTTTTATACTTAC and reverse primer 5′- TACGCCAATAAAGAATTGAGCTG at a 25 μl volume. For PCR reaction, 200 ng genomic DNA was amplified using the optimized protocol, in which the DNA was denatured for 5 min at 95 °C and amplified over 6 cycles of 10 s at 95 °C, 20 s at 62 °C (decreasing 1 °C for each cycle) and 80 s at 72 °C, followed by 40 cycles of 10 s at 95 °C, 20 s at 56 °C and 80 s at 72 °C, finally annealed for 5 min at 72 °C. Genotypes were manually assigned based on Chromas software (version 2.23).

### Subjects for association study

The study sample consisted of 19 bipolar disorder patients (nine males and ten females, 37.7 ± 13.1 years) and 17 healthy subject controls (seven males and ten females, 31.8 ± 10.5 years), which were type I or type II bipolar disorder subjects recruited from the Shatin Hospital, Hong Kong. The samples of four members of the 16-year-old boy’s family were also included in this study.

They were all Chinese of Han ethnic origin. All subjects were required to conduct the CB-SCID-I/P interview. Bipolar disorder patients suffering from mental retardation or organic brain syndrome (i.e. dementia) were excluded. All subjects’ written informed consents were obtained after a comprehensive and detailed description of this study. This study was approved by the Joint Chinese University of Hong Kong–New Territories East Cluster Clinical Research Ethics Committee.

### Statistical analysis

Comparisons of continuous and categorical variables were accomplished by *t* test and chi-square test respectively using SPSS program (version 20.0). The genotypic and allelic frequencies between the bipolar disorder patients and healthy controls were compared by genotypic model and allelic model respectively using case–control association test in UNPHASED program (Dudbridge 2006, 2008). Hardy-Weinberg equilibrium (HWE) tests were used to examine the genotypic distribution of SNPs in samples. Linkage disequilibrium (LD) between SNPs was calculated in Haploview program using r^2^ algorithm (version 4.2) (Wigginton et al. 2005). LD block plots of *DGKH* gene was generated based on the population of Han Chinese genotype data downloaded from the HapMap project (Data Phase III/Rel#2, Feb09). The association study of 2-marker haplotypes with bipolar disorder was also performed in Haploview (Barrett et al. 2005). Moreover, given the possible error produced by partially dependent SNPs and small sample size in this study, 2000 permutation tests were used to run corrections for those preliminary significant tests. P-values after permutation tests less than 0.05 were considered statistically significant.

### Human and animal rights and informed consent

All procedures followed were in accordance with the ethical standards of the responsible committee on human experimentation (institutional and national) and with the Helsinki Declaration of 1975, as revised in 2000 (5). Informed consent was obtained from all patients for being included in the study.

## Results

### Hypomania symptoms of a 16-year-old bipolar Chinese boy

The boy was well until after his 16th birthday. He had a sudden onset of a hypomania episode precipitated after a stressful situation at school. On the day of manifestation of symptoms, he had consumed some alcohol (approximately 200 ml of rice wine) at a ceremony. After the event, he suffered from insomnia and pressure of speech. A few days later, he had grandiose ideas and announced to run for the president of the student union. After 5 days of sleep deprivation, he could not attend school and stayed home to rest, reading and playing computer games sometimes. He lost his appetite, in addition to sleeplessness, and started talking to himself, mostly about how to play certain computer games, and talked to imaginary persons about various plots that are not comprehensible to others, which was indicative of auditory/visual hallucination. On the 6th day, he went hiking and was noticed to pick up heavy objects while hiking and made loud noises by banging them on the ground or squeezing coke bottle as if to ease his frustration.

On hospitalization, he was screened for organic causes and found to be negative for infection after tests with blood and spinal fluid (antibody against N-methyl-D-aspartate receptor (NMDAR) was also negative in spinal fluid). No brain injury and no brain tumor were obvious from MRI brain, X-ray and a computerized tomography scan. His laboratory findings were mostly within normal range. After nearly 3 weeks, his conditions stabilized with Valproate Controlled Release 400 mg once a day and Olanzapine 20 mg once a day, and his weight increased from 51.6 kg to 59 kg half a year after medication without any increase in height (1.63 m).

**Fig. 2:**
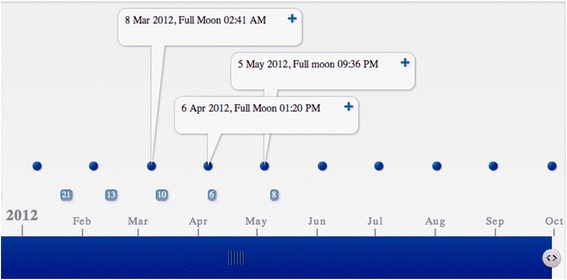
Episodes of hypomania as a function of time plotted by time toaster (a freely available web tool). The time of a full moon is represented by a *filled spot* while an episode of hypomania is represented with an *unfilled spot*, with various sizes scaled to the duration of the episodes. Supplementation of phosphatidylcholine was initiated on May 28, 2012, and no hypomania episode was observed after that date for five months (from mid-May to mid-Oct, 2012) and following nine months (data not shown)

However, even several months after he was discharged from the hospital, the medication of valproate and olanzapine did not prevent monthly episodes of hypomania and sleeplessness (which happened three alternate nights every month, mostly 3 days after the full moon) that occurred at monthly interval for 5 months in 2012 (From mid-Jan to mid-May, see Fig. 2). His hypomania features include elevated mood, participation in risky behaviors such as making 10 km (walking or biking) trips in deserted streets on his own without informing anyone, going through a public park, at 3 or 4 am to school and playing basketball with complete strangers much older than himself. He also suffered from distractibility, spent money freely and had increased goal directed activities during his monthly cycles of hypomania.

### Beneficial effect of phosphatidylcholine supplementation for the 16-year-old Chinese boy’s hypomania symptoms

When the phosphatidylcholine supplementation was initiated in mid-May, in addition to the valproate and olanzapine medications, after five months’ hypomania episode (from mid-Jan till mid-May, 2012) the boy’s laboratory findings were mostly normal, including hemogram, WBC differential count, blood glucose tests, thyroid function test, C-reactive protein, RA Factor, liver function tests, renal function test, uric acid level, calcium, phosphate, iron levels, tumor markers (alpha fetal protein and CEA). Although total cholesterol, HDL, and LDL were within normal range (4.1, 1.53 and 2.22 mmol/L respectively), the total cholesterol/HDL ratio was slightly lower than that of the normal range (2.7, normal range: 3.0-6.7).

In the months following the initiation of phosphatidylcholine supplementation, no hypomania symptoms and insomnia were observed after the full moon, which means that there are no nights that he cannot sleep throughout the night and the disappearance of those hypomania features before supplementation (Fig. 2). The boy has been taken off all his medication after half a year of supplementation and has been symptoms free for five months (mid-May till mid-Oct, 2012, Fig. 2) and the following nine months (mid-Oct till mid-July, 2013, data not shown) since he initiated the supplementation.

### Association study with bipolar disorder in Chinese

The “no call” signal- rs9532989 in sequencing data and its nearby SNPs in the same LD block with rs9532989 in the first intron of *DGKH* were chosen to carry out the association study of bipolar disorder in an age and gender-matched Hong Kong Chinese cases and controls.

**Fig. 3:**
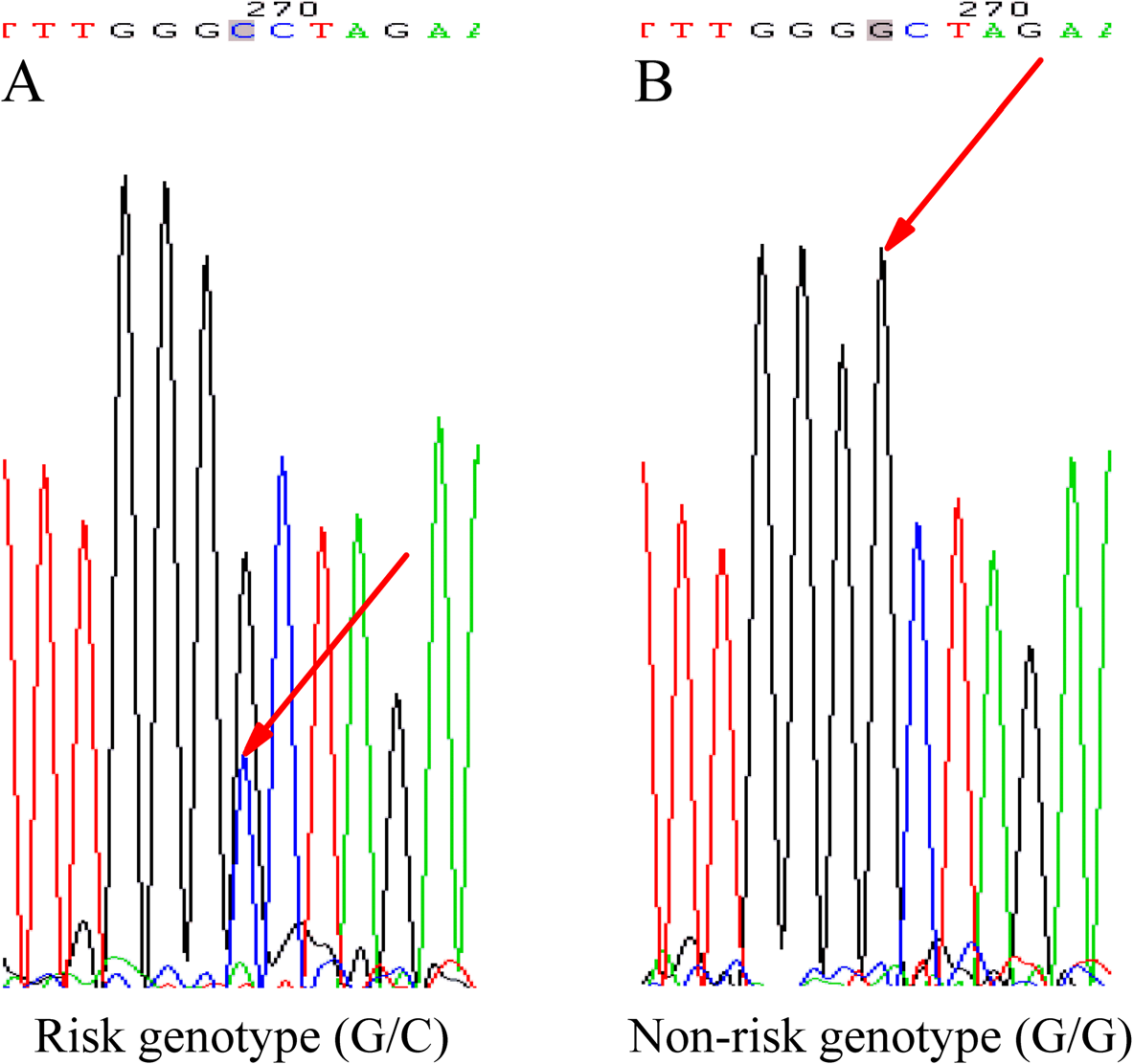
DNA sequence analyses of rs77072822 polymorphism in the 16-year-old boy with bipolar hypomania (**a**) and his 19-year-old healthy sister (**b**)

The call rate of genotyping was 100 % complete for the bipolar disorder patients and healthy controls. There is no difference between cases and controls group in gender (*p* = 0.749) and age (*p* = 0.147). In allelic analyses, we did not find any polymorphism in two SNPs – rs17519207 (*T* = 1.0, *C* = 0.0) and rs79738935 (*C* = 1.0, *T* = 0.0), which were therefore non-informative. The sequencing results of all samples in the remaining eight SNPs are shown in Table 1. All the distribution of the eight SNPs in bipolar disorder patients and healthy controls were in accordant with the Hardy-Weinberg equilibrium (*P* > 0.276). In single-marker association analysis, we found that a SNP rs77072822 was significantly associated with bipolar disorder in allelic analyses (*p* = 0.011) and genotypic analyses (*p* = 0.003). And these associations remained significant after 2000 permutation tests (*p* = 0.045 for allelic analyses and *p* = 0.025 for genotypic analyses) (Table 1). Moreover, the frequency of GC in rs77072822 was over-represented in bipolar disorder patients comparing with the healthy controls, which indicated that GC might be a risk genotype to bipolar hypomania. Coincidentally, the DNA sequence of the 16-year-old boy is the risk genotype (G/C) in rs77072822 (Fig. 3a), whereas his healthy sister carries the non-risk genotype (G/G) (Fig. 3b). Furthermore, association analyses of all two-maker haplotypes containing rs77072822 with bipolar disorder were carried out. And we found that all of the haplotypes were significantly associated with bipolar disorder after 2000 permutation tests (*P* < 0.043) (Table 2). Besides, neither rs9532988 nor rs9532989 was found to be associated with bipolar disorder in this sample (Nominal *p*-value = 0.537 and 0.946, respectively) which was consistent with Zeng’s findings, although the two SNPs were associated with bipolar disorder in a sample of National Institute of Mental Health in Baum’s study (Fig. 1).

**Table 1:** Single-marker analysis of eight SNPs^a^ with bipolar disorder

	Associated allele	Frequency		Allelic analyses		Genotypic analyses	
SNP ID		Case	Control	*P* value (Nominal)	*P* value (Perm) ^b^	*P* value (Nominal)	*P* value (Perm)
rs7990452	A	0.447	0.412	0.761	-	0.956	-
rs9532987	A	0.447	0.412	0.761	-	0.956	-
rs9562372	C	0.500	0.471	0.803	-	0.388	-
rs77072822	C	0.237	0.029	0.011*	0.045*	0.003**	0.025*
rs9532988	A	0.947	0.912	0.553	-	0.537	-
rs9532989	G	0.474	0.471	0.979	-	0.946	-
rs4142112	C	0.474	0.382	0.435	-	0.700	-
rs4142111	C	0.974	0.382	0.128	-	0.347	-

^a^ *SNPs* single nucleotide polymorphisms, ^b^ *P* value (Perm): *P* value after 2000 permutation tests; *P < 0.05; **P < 0.01

**Table 2:** Association analyses between two-marker models with rs77072822 and bipolar disorder

		Frequency		*P* value^a^
Block	Haplotype	Case	Control	(Perm)
rs77072822-rs7990452	C-T	0.237	0.029	0.038*
rs77072822-rs9532987	C-G	0.237	0.029	0.034*
rs77072822-rs9562372	C-C	0.237	0.029	0.043*
rs77072822-rs9532988	C-A	0.237	0.029	0.012*
rs77072822-rs9532989	C-T	0.237	0.029	0.019*
rs77072822-rs4142112	C-G	0.237	0.029	0.036*
rs77072822-rs4142111	C-C	0.237	0.029	0.012*

^a^ *P* value (Perm): *P* value after 2000 permutation tests, *p < 0.05

## Discussions

In this study, we have reported on a 16-year-old Chinese boy with bipolar hypomania symptoms who was initially psychotic, and upon treatment with medication, responded well and stabilized. However, he still suffered from monthly episodes of insomnia accompanied by hypomania for 5 months despite adherence to medication. After that, he was put on a supplement of one capsule mainly containing 600 mg phosphatidylcholine. In the month following the initiation of phosphatidylcholine supplementation, no hypomania and insomnia was observed after the full moon and all hypomania symptoms disappeared. He has been taken off all his medication after half a year of supplementation and has been symptoms free for approximately one year. This finding was consistent with Chen’s report, in which they found that the choline level, considered as sex-specific urinary metabolite biomarkers for diagnosing male and female bipolar disorder patients, was decreased in bipolar disorder male subjects (Chen et al. 2014). The decline of phosphatidylcholine might be caused by the unbalance of metabolism of DAG and PA, in which DGK enzymes phosphorylate DAG into PA when receptors in phosphoinositide pathway receiving activation. This hypothesis suggested that DGK enzymes are likely to play a vital role in the pathogenesis of bipolar hypomania symptoms.

Diacylglycerol kinase eta (*DGKH*), encoding an important member of DGK enzymes family, has been reported associating with bipolar disorder in a genome-wide association study (GWAS) and replicated in Chinese Han population (Baum et al. 2008; Zeng et al. 2011). Meta-analyses in metamoodics database also compiled results of several GWAS studies and confirmed the association of DGK enzymes with bipolar disorder. Moreover, the role of *DGKH* in mental disorder was further confirmed from data obtained in animal studies. Mapping of different strains of mice found that a region in chromosome 14 (which contains *DGKH*) is associated with the midsagittal area of the corpus callosum (MSACC), which has been associated with a number of cognitive and behavioral phenotypes, including bipolar disorder, obsessive-compulsive disorders, psychopathy, suicidal tendencies, schizophrenia, autism, and attention deficit hyperactivity disorder (Newbury and Rosen 2012).

In this study, for the first time the association analysis showed that GC in rs77072822 is over-represented in bipolar disorder patients indicating that GC might be a risk genotype to bipolar hypomania. Thus, we assume that the missed call of the boy different from his three family members in rs9532989 might be due to the mutation in rs77072822 as these two polymorphisms have a very close physical position in chromosome and are locate in the same linkage disequilibrium block (Fig. 1).

This SNP marker that we found to be associated with bipolar disorder is a novel marker that has not been reported in previous studies, although it is located in the same region, i.e. the first intron of *DGKH,* which was mostly reported to be associated with bipolar disorder (Baum et al. 2008; Zeng et al. 2011). Such differences in marker SNP between our study and the previous studies could be due to differences in the risk mutation located in a same LD block for the different populations, or that the risk allele arose separately on different genetic background in different regions. In addition, we found that the 16-year-old boy carries the risk genotype (G/C) in rs77072822, whereas his 19-year-old healthy sister has the non-risk genotype (G/G). Together, these findings could provide a possible explanation at the genetic level why the 16-year-old boy responded well to supplementation of phosphatidylcholine. However, this possible hypothesis should be supported by more evidence at either the mRNA or the protein level.

One limitation of our study is that the effect could be compounded by the presence of vitamin B complex in the supplement; however, earlier supplementation of vitamin B complex did not alleviate his hypomania and insomnia. Further double blind, randomized, placebo-controlled clinical trials should be carried out to test the hypothesis that subjects with the risk genotype of *DGKH* respond better to phosphatidylcholine supplementation than those observed with the non-risk genotype. In addition, the small sample size of association study might affect the validity of significant association, which suggests that the data in this study should be interpreted with caution; nevertheless, the association of rs77072822 with bipolar disorder remained significant after 2000 permutation tests. Although we reported that the hypomanic symptoms and the insomnia were observed after the full moon, not much is known about the scientific meaning of this association despite extensive searching of the literature, thus it is likely that the timing of the episode after the full moon is merely a chance event, i.e. the hypomanic episode occurred regularly in this case and it just happened to fall after the full moon.

In summary, the 16-year-old boy appears to have benefited from the supplementation with phosphatidylcholine and recovered from hypomania symptoms. He carries a risk genotype (G/C) in rs77072822 which lies in the first intron of *DGKH* gene that was mostly reported to be associated with bipolar disorder. Thus, this finding is consistent with the hypothesis that alleviating the phosphatidylcholine deficiencies might accompany with the risk variant of *DGKH*, Personalized genomics might improve the efficacies of such supplementation and help in the design of new treatment strategies for bipolar disorder.
